# Survey of Candidate Genes for Maize Resistance to Infection by *Aspergillus flavus* and/or Aflatoxin Contamination

**DOI:** 10.3390/toxins10020061

**Published:** 2018-01-31

**Authors:** Leigh K. Hawkins, Marilyn L. Warburton, Juliet D. Tang, John Tomashek, Dafne Alves Oliveira, Oluwaseun F. Ogunola, J. Spencer Smith, W. Paul Williams

**Affiliations:** 1 USDA ARS Corn Host Plant Resistance Research Unit, Mississippi State, MS 39762, USA; marilyn.warburton@ars.usda.gov (M.L.W.); spencer.smith@ars.usda.gov (J.S.S.); paul.williams@ars.usda.gov (W.P.W.); 2 USDA Forest Service, Forest Products Laboratory, Starkville, MS 39759, USA; julietdtang@fs.fed.us; 3 Integrated Micro-Chromatography Systems LLC, Irmo, SC 29063, USA; jt.posterboy@hotmail.com; 4 Department of Biochemistry, Molecular Biology, Entomology and Plant Pathology, Mississippi State University, Starkville, MS 39762 USA; dao59@msstate.edu; 5 Department of Plant and Soil Sciences, Mississippi State University, Starkville, MS 39762, USA; ofo6@msstate.edu

**Keywords:** maize, *Zea mays* L., *Aspergillus flavus*, aflatoxin, candidate genes, Quantitative trait loci associated with resistance to *Aspergillus flavus* and/or aflatoxin production can be quite large and are comprised of thousands of genes, thus making it difficult to determine which one(s) may be most relevant to pursue in breeding for improved resistance. The authors examined maize mapping populations for association of polymorphisms within 195 candidate genes that may be involved in resistance. Many of the identified candidates genes offer clues to key metabolic and/or enzymatic pathways that may have a significant effect on reducing fungal infection and/or aflatoxin accumulation.

## Abstract

Many projects have identified candidate genes for resistance to aflatoxin accumulation or *Aspergillus flavus* infection and growth in maize using genetic mapping, genomics, transcriptomics and/or proteomics studies. However, only a small percentage of these candidates have been validated in field conditions, and their relative contribution to resistance, if any, is unknown. This study presents a consolidated list of candidate genes identified in past studies or in-house studies, with descriptive data including genetic location, gene annotation, known protein identifiers, and associated pathway information, if known. A candidate gene pipeline to test the phenotypic effect of any maize DNA sequence on aflatoxin accumulation resistance was used in this study to determine any measurable effect on polymorphisms within or linked to the candidate gene sequences, and the results are published here.

## 1. Introduction

The projected worldwide production of corn (*Zea mays* L.) for 2017/18 is 1031.86 million metric tons [1]. The United States provides more than half of the total supply of corn to the world market [2]. In addition to human consumption, corn has many uses as animal feed and for other industrial purposes. One of the phytopathogens that infects corn is the opportunistic, fungal saprophyte, *Aspergillus flavus*. *A. flavus* produces toxic secondary metabolites known as aflatoxins, which cause a range of deleterious effects in humans and animals, including hepatocellular carcinoma, liver toxicity and growth impairment [2]. The economic impact of aflatoxins is derived from crop and livestock losses as well as regulatory control to minimize the risks to animal and human health [2] Breeding for resistance to aflatoxin accumulation and ear rot caused by *A. flavus* in maize has been hampered by a low heritability, environmental effects, and the highly quantitative nature of the trait [3].

Quantitative trait loci (QTL) have been reported for resistance to aflatoxin accumulation [4]; however, the QTL intervals are generally too large for marker-assisted selection and no QTL explains more than 20% of the phenotypic variation in any given mapping experiment [5]. Many projects have taken a difference approach and identified candidate genes for resistance to aflatoxin accumulation or *A. flavus* infection and growth in maize using genetic mapping, genomics, transcriptomics and/or proteomics studies [6,7,8,9,10]. However, only a small percentage of these candidates have been validated under field conditions and their relative contribution to resistance, if any, is unknown [11,12]. The United States Department of Agriculture Agricultural Research Service (USDA-ARS) Corn Host Plant Resistance Research Unit utilizes a candidate gene-testing pipeline that consists of steps for identifying, testing and verifying the statistical association or genetic linkage of any maize gene sequence with resistance to aflatoxin accumulation [3]. The pipeline includes four QTL-mapping populations and one association-mapping panel, all of which have been fully phenotyped over multiple years and locations for aflatoxin-accumulation resistance and associated phenotypes. The QTL populations have been genotyped with between 118 to 225 genetic markers, and the association panel via genotyping by sequencing. Hundreds of genes identified as possible resistance candidates in the literature or in our Corn Fungal Resistance Associated Sequences database (CFRAS-DB, [10]) of candidate gene information have been put into the candidate-gene testing pipeline. Single nucleotide polymorphisms (SNPs) and insertion or deletion (InDel) polymorphisms within each gene that map to the correct genomic location, or closely linked simple sequence repeat (SSR), SNP and InDel markers, were tested for phenotypic effect on aflatoxin-accumulation resistance, and results are presented here.

In previous research conducted by this unit, metabolic pathways [9], chitinase family genes [11] and lipoxygenase family genes [12], which are involved in maize aflatoxin-accumulation resistance, have been identified and characterized This manuscript provides an overview of all candidate genes that have been screened by this laboratory via candidate-gene association analysis or QTL mapping to date.

## 2. Results

### 2.1. Candidate Gene Functions

To date, a total of 195 candidate genes have been evaluated. These genes occur in various locations throughout the maize genome (Table S1). The Kyoto Encyclopedia of Genes and Genomes (KEGG) PATHWAY Database [13,14] was used to characterize genes that were involved in different metabolic processes and pathways. Upon contact with pathogens or other elicitors, a variety of responses are activated by the plant such as changes in primary metabolism, ion fluxes, phosphorylation/dephosphorylation of proteins, production of signal molecules and generation of reactive oxygen species. This ultimately leads to the regulation of gene expression and induction of defense responses including strengthening of the cell wall and accumulation of pathogenesis-related (PR) genes [15]. The status of metabolites including sugars and amino acids is crucial, since they often serve as substrates and as signaling molecules for responses to environmental stress factors and/or interactions with pathogens [16].

Sixty-five of the candidate genes are involved with genetic information processing via transcriptional or translational regulation, and/or post-translational modification. These genes may also be involved with replication, repair, folding, sorting and/or degradation of nucleic acids and proteins. Transcription factors (TF) regulate responses to various plant stresses in multiple and complex signaling pathways and play a role in plant–pathogen interactions. The cross regulation of the WRKY TFs on the list may ensure rapid, efficient defense signaling in coordinating the response of an affected plant to biotic and abiotic stresses [17,18,19,20,21]. Two *R2R3-MYB* proteins (GRMZM2G166337 and GRMZM2G160840 [22]) are of interest, because MYB transcription factors are involved in the regulation of secondary metabolism and the response to stress conditions [23,24,25]. GRMZM2G136910, abscisic acid stress ripening1, plays multiple roles as a transcription factor and a chaperone-like protein [26,27]. Other genes of interest within this group include GRMZM2G165901, the glycine-rich RNA binding abscisic acid inducible protein which regulates several genes involved in water-stress tolerance in maize. The high-mobility group (HMG) proteins [28] are involved in the regulation of transcription and recombination. Squamosa promoter-binding protein-like (SPL) genes [29] are important in plant growth and development, gibberellins signaling, and response to fungal toxins. In eukaryotes, gene expression can be regulated by RNA interference at the post-transcriptional level or chromatin modification at the transcriptional level [30]. The Dicer-like protein (GRMZM2G024466) may be involved in processing dsRNA into smaller siRNA for gene silencing [30]. The ROP guanine nucleotide exchange factor (GRMZM2G147780) plays a role in the molecular transduction of extracellular signals. Many of the remaining genes in this category are involved with post-translational modification, protein turnover, protein transport and/or serve as chaperones. Other genes are involved in response to abiotic and/or biotic stress, e.g., heat-shock proteins [31], and protein kinases.

Another large portion of these candidate genes on this list are involved in carbohydrate metabolism. The carbohydrate status of the host plays a role in the defense and in the general metabolism of the plant. In addition to serving as nutrients, sugars may act as osmoprotectants or assist in responses to abiotic stresses [32] or serve as signal molecules to regulate gene expression [15]. Included in this group are chitinolytic genes and other beta-hexosaminidases that are part of the chitin degradation II pathway (PWY-6902). Several of the chitinases also play a role in signal transduction (KOG4742) as part of the mitogen-activated protein kinases (MAPK) signaling pathway (ko04016). The suggested primary function of induced expression of plant chitinases, acting alone or in conjunction with β-1,3-glucanases or other antifungal compounds, is defense against fungal pathogens [4,33,34,35,36] In a previous study, Hawkins et al. [11] characterized maize chitinases for their effect on aflatoxin production and accumulation. Fifteen of the candidate genes are involved in the trehalose metabolism either as part of the biosynthetic pathway (TRESYN-PWY) or the degradation of trehalose (PWY0-1182). Plants have intricate sugar-signaling networks to maintain energy status regardless of growth status, and the trehalose pathway appears to play a role with effects on flowering, embryogenesis, biomass, and abiotic/biotic stress tolerance [16,37] Ten of the remaining genes in this group are involved with carbohydrate degradation: sucrose degradation (PWY-621); homogalacturonon (pectin) degradation (PWY-1081); UDP-glucose biosynthesis (PWYQT-4437); glycolysis, cellulose biosynthesis (PWY-1001); the Calvin cycle and gluconeogenesis. The remaining genes are involved with transport.

Another subset of the candidate gene is involved with environmental information processing, signal molecules, stress responses and other cellular processes including amino-acid metabolism and the biosynthesis of secondary metabolites. These play a role in membrane transport, signal transduction and other signaling interactions. Cellular responses to external and internal stimuli require the regulation of the flow of compounds and the relay of signaling events. Many of the kinases identified play a role in a variety of developmental and defense-related processes via protein phosphorylation [38]. Calmodulins and calcineurin-B-like proteins serve as Ca^2+^ sensors in the complex, interconnected signaling pathways [39]. Two pathogenesis-related PR-1 proteins (GRMZM2G465226 and AC205274.3_FG) are part of the MAPK signaling pathway and members of the cysteine-rich secretory protein (CRISP) family [40]. This section also includes several stress-related resistance-associated proteins (RAPS) that possibly play a role in host defense to *Aspergillus flavus* infection [41].

*GRMZM2G176977*, *GRMZM2G151440*, *GRMZM2G052991*, *GRMZM2G108416* and *GRMZM2G152470* play a role in amino-acid metabolism. These genes are primarily in the biosynthesis of the branched chain amino acids (BCAA), leucine, isoleucine and valine. In addition to being the building blocks of protein, amino acids (and the BCAA in particular) play roles in the development and growth and stress responses of plants. The metabolism of amino acids is required for the biosynthesis of several plant-protective products and may play a role in modulating the activity of other defense mechanisms [42]. Methionine is a precursor of ethylene and isoleucine is required for the activation of jasmonic acid [43].

Several of the genes encode enzymes that are required for the biosynthesis of secondary compounds. The induction of many of these genes is necessary to launch diverse plant defensive mechanisms; they often serve as substrates or secondary messengers for other molecular and physiological responses [8]. Cytochrome P450 enzymes are involved in the biosynthesis of hormones, phytoalexins, and xenobiotics [44]. *GRMZM2G085661* (Bx2) as part of the DIMBOA pathway has been associated with aphid and fungal resistance [45,46]. The *s*-adenosyl-l-methionine-dependent methyltransferases (SAM-Mtases) are involved in the production of many secondary products such as lignin, flavonoids and phytoalexins [47]. Many of these genes are involved in the redox homeostasis [48]

Twenty-one genes from the list are involved in fatty acid and/or lipid metabolism. This group includes 7 lipoxygenase (LOX) genes. LOX genes serve different functions in the plant including growth and development, pest resistance, senescence and/or wounding responses. LOX genes in relation to maize aflatoxin resistance have been characterized by this lab [12]. The remaining genes in this group play roles in beta oxidation, jasmonic acid biosynthesis, choline biosynthesis and/or cAMP signaling. Metabolic pathways associated with aflatoxin accumulation and resistance were identified by Tang et al. [9]; these include PWY-735 jasmonic acid biosynthesis, PWY-5136 fatty acid β-oxidation II (core pathway) [49], and PWY-5409 divinyl ether biosynthesis II.

Five of the candidate genes are uncharacterized and of unknown function. *GRMZM2G053140*, *GRMZM2G165601*, *GRMZM2G108619* and *GRMZM2G331766* were identified via genome-wide association study (GWAS) analysis [9,50] as being associated with resistance to aflatoxin contamination. *GRMZM2G166166* (TC462902) was identified via transgenic maize analysis [51].

### 2.2. Significant Associations or Linkages to Phenotypic Effects

Of the 195 candidate genes in the study, 102 contained SNPs or InDels that were associated with resistance to *A. flavus* contamination and/or aflatoxin accumulation in a candidate-gene association analysis at *p* < 10^−3^, of which, 39 were still significant at the *p* < 10^−6^ level (Table S1). There were 68 intervals linked to a QTL in one or more QTL-mapping population with a logarithm of the odds (LOD) score greater than 3.5 containing one or more of the candidate genes, as several of the genes were linked to a degree that we could not determine which (or if both) were causing the QTL effect seen. There were only 59 genes neither linked to QTL in at least one mapping population nor associated with aflatoxin accumulation in the GWAS panel (Table S1). Thirty four of the candidate genes were both within a QTL interval and associated with aflatoxin accumulation. These results must be taken with some caution, however, since an average of 6.7% of the maize genome are covered by QTL for aflatoxin accumulation with a LOD greater than 3.5 in the four mapping populations (ranging from 1.2% of the genome of MpNC to 12.5% of the genome in MpB; Supplemental File). To generate an idea of the number of associations seen by coincidence from the same GWAS data, a set of 300 SNPs was chosen at random and run through the general linear model (GLM) analysis. Ten were found to be associated with aflatoxin accumulation at *p* < 10^−3^; but, none were associated at a higher significance (data not shown). Therefore, a small percentage of the associations and linkages found here may be just coincidental; however, there are more than expected by random chance, and thus most associations and linkages will be statistically valid. This indicates that the publications from which they were originally chosen were finding biologically relevant genes for resistance to aflatoxin accumulation.

Just over 70% of the genes in this study were associated or linked with an effect on reducing aflatoxin accumulation in one or more study. These genes were found within all the KEGG pathway annotation categories discussed here, and in four of the six categories the linked or associated genes accounted for over 65% of the total genes. Genes in categories having to do with response to the environment and, in particular, to stress, or the biosynthesis of secondary compounds, over 80% of the genes were linked or associated with resistance. This indicates that plant activities corresponding to each of the KEGG categories may be contributing to reducing the levels of aflatoxin found in infected maize ears, and that mechanisms to sense the threat, and production of compounds to fight it, may be most important.

## 3. Discussion and Conclusions

Resistance to infection by *A. flavus* and/or accumulation of aflatoxin is a quantitative trait. While none of the genes identified here are highly significant, and none explain a high percentage of the phenotypic variation of the trait (data not shown), many of the identified candidate genes offer clues as to their role in the mechanics of resistance to fungal infection and/or toxin accumulation. Several genes may act synergistically to provide resistance due to crosstalk between transcription factors and other candidate genes [52]. A number of the identified candidate genes are involved in primary plant metabolism: carbohydrate metabolism, energy metabolism, and lipid metabolism. These metabolic pathways are all necessary for plant growth, differentiation and responses to environmental cues [53]. Evaluation of their function as part of a pathway may lead to the identification of key metabolic and/or enzymatic pathways that may have a significant effect in reducing aflatoxin accumulation or fungal infection [4]. Although many of the genes are not involved in annotated metabolic plant pathways, they may still play roles in maize resistance e.g., by providing structural barriers to fungal penetration or by modifying the environment by inhibiting other pathways [4]. Given that *A. flavus* is primarily a saprophyte and its more deleterious effects are highly dependent on the environment, narrowing the gene(s) that are required for resistance is a challenge. By continuing to investigate candidate genes of interest and pursuing more evidence with gene expression with genotypes of varying resistance, the interaction between the host and the pathogen can possibly be regulated to minimize fungal infection and/or toxin production.

## 4. Materials and Methods

### 4.1. Classification of Candidate Genes

**Figure 1:**
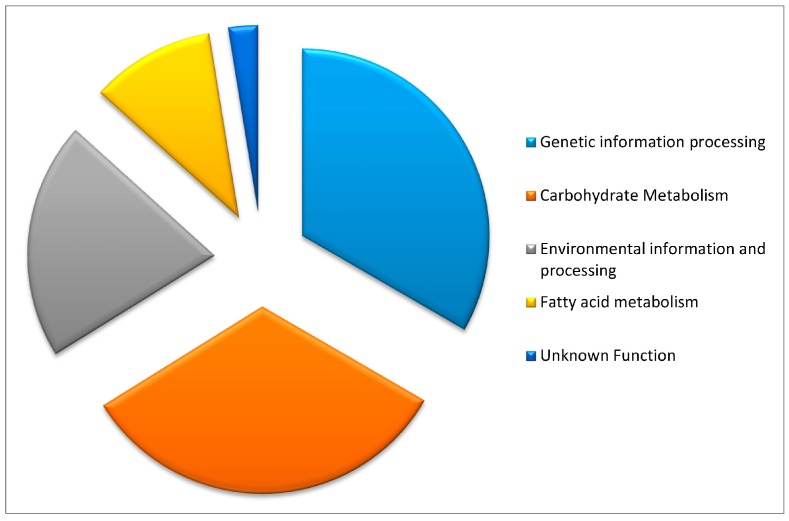
Proportion of gene candidates by Kyoto Encyclopedia of Genes and Genomes (KEGG) orthology.

These candidate genes were initially identified by multiple investigators via various methods including proteomics, GWAS analysis, microarray analysis and the CFRAS database [4,6,8,10,50,54]. As a means of organizing the genes, broad groups (Figure 1) were made using the available Gene Ontology information from Gramene [55], Uniprot [56,57], Phytozome [58], the Pfam database [59], the Poaceae Intronless Genes Database [60] and KEGG [61]. A table of all 195 candidate genes can be found in the Table S1.

### 4.2. Materials and Methods

The QTL mapping populations used for verification of phenotypic effects of each candidate gene consisted of four F_2:3_ linkage mapping families. These mapping populations have been characterized and published previously. The mapping populations were derived from initial crosses between the following pairs of parents: Mp313E (resistant to aflatoxin accumulation) and Va35 (susceptible) [62], Mp313E and B73 (susceptible) [63]; Mp715 (resistant) and T173 (susceptible) [64]; and Mp717 (resistant) and NC300 (susceptible) [65]. F_1_ plants of each initial cross were selfed to create F_2_ plants, individual seeds of which were grown and selfed to create F_2:3_ families. DNA from each F_2_ plant was used for genotyping using SNPs, SSRs or restriction fragment length polymorphisms (RFLP) markers, and linkage mapping. F_2:3_ families were grown in replicated field tests in multiple environments (see individual mapping references for details on the phenotyping of each population). Briefly, 10 plants of each family were individually inoculated with a 3.4-mL suspension of 3 × 10^8^ conidia of *Aspergillus flavus* strain NRRL 3357 (ATCC #200026) using the side-needle technique 7 d after mid-silk [66,67]. Bulked grain samples from each family were dried, shelled, ground and tested for aflatoxin concentration in 50 g samples of ground grain from each plot using the VICAM AflaTest^®^ (VICAM, Watertown, MA, USA), according to the manufacturer’s instructions.

Genetic linkage analyses were conducted as in Hawkins et al. [11]. SNP markers from a maize subset of KASP assays from LGC Genomics were tested for polymorphism between the parents of all mapping populations. When polymorphisms were found, the markers were run on the entire mapping population to test the effect of each gene on the phenotype. When polymorphic SNPs could not be identified in any mapping population for a given gene sequence, SSR markers within 10,000 Kb were used to test the effect of the region. Flanking markers were used, when possible, and in some cases, a marker of unknown physical location, but very close genetic location, or an SSR further than 10,000 Kb, was used as one of the flanking markers. In both of these cases, the second flanking marker was always within 10,000 Kb. Mapping was done using the JoinMap mapping software (version 4, Kyazma BV, Wageningen, Netherlands, 2006) [68]; and linkage groups were constructed using the maximum likelihood (ML) mapping method. Composite interval mapping (CIM) was performed using QTL Cartographer version 2.5 (North Carolina State University, Raleigh, NC, USA, 2012) [69] as described by Warburton et al. [65]. To estimate the 0.05 significance threshold for QTL, 1000 permutations were performed with each dataset and across all datasets [70]. Mapping was done for each year, each location, and across both locations and years, where each population was phenotyped.

The aflatoxin association-mapping panel consisted of 282 diverse inbred lines, which have been characterized as described previously [50]. Briefly, testcrosses were formed with Va35, a susceptible, southern-adapted inbred line of the non-stiff stalk heterotic pattern, and grown in seven environments. Plants were inoculated and phenotyped as described in the QTL mapping populations, above. Proc GLIMMIX from the SAS statistical software package was used to calculate least-squares means (LSMEANS) of aflatoxin levels using a generalized linear mixed model (GLMM). Both log-transformed and untransformed (but not quite normal) data were used in the association analysis. Genotyping of the 282 entries in the panel was done via genotyping by sequencing (GBS) according to [71]. A data subset consisting of 2000 SNPs was used to calculate population substructure using Structure 2.2 [72], and a kinship matrix using PowerMarker v. 3.25 [73] to correct for population substructure during association analysis using the Mixed Linear Model of TASSEL 3.0.1 [74]. SNPs within the reported genetic sequences of the candidate genes (Table S1), or within a ± 15 Kb window, were extracted from the GBS dataset for association analysis and are listed in the Table S1. SNPs were filtered to remove those with a minor allele frequency of less than 5%. The transcription level and tissue specificity of selected candidate genes was analyzed from the B73-derived gene atlas [75].

## Supplementary Materials

The following are available online at http://www.mdpi.com/2072-6651/10/2/61/s1, Table S1: Descriptive Information for Candidate Genes Associated with Resistance to Kernel Infection by *Aspergillus flavus* and/or aflatoxin accumulation.
